# Can food vouchers improve nutrition and reduce health inequalities in low-income mothers and young children: a multi-method evaluation of the experiences of beneficiaries and practitioners of the Healthy Start programme in England

**DOI:** 10.1186/1471-2458-14-148

**Published:** 2014-02-11

**Authors:** Alison McFadden, Josephine M Green, Victoria Williams, Jenny McLeish, Felicia McCormick, Julia Fox-Rushby, Mary J Renfrew

**Affiliations:** 1 Research Fellow, College of Medicine, Dentistry and Nursing, University of Dundee, 11 Arlie Place, Dundee DD1 4HJ, UK; 2 Department of Health Sciences, University of York, Heslington, York YO10 5DD, UK; 3 Food Matters, Brighthelm Centre, North Road, Brighton BN1 1YD, UK; 4 4 Claremont Road, Tunbridge Wells, Kent TN1 1SZ, UK; 5 Health Economics Research Group (HERG), Brunel University, Uxbridge, Middlesex UB8 3PH, UK; 6 College of Medicine, Dentistry and Nursing, University of Dundee, 11 Arlie Place, Dundee DD1 4HJ, UK

**Keywords:** Food subsidy programme, Food vouchers, Healthy Start, Low-income families, Maternal and young child nutrition, Fruit and vegetable intake, Nutritional inequalities

## Abstract

**Background:**

Good nutrition is important during pregnancy, breastfeeding and early life to optimise the health of women and children. It is difficult for low-income families to prioritise spending on healthy food. Healthy Start is a targeted United Kingdom (UK) food subsidy programme that gives vouchers for fruit, vegetables, milk, and vitamins to low-income families. This paper reports an evaluation of Healthy Start from the perspectives of women and health practitioners.

**Methods:**

The multi-method study conducted in England in 2011/2012 included focus group discussions with 49 health practitioners, an online consultation with 620 health and social care practitioners, service managers, commissioners, and user and advocacy groups, and qualitative participatory workshops with 85 low-income women. Additional focus group discussions and telephone interviews included the views of 25 women who did not speak English and three women from Traveller communities.

**Results:**

Women reported that Healthy Start vouchers increased the quantity and range of fruit and vegetables they used and improved the quality of family diets, and established good habits for the future. Barriers to registration included complex eligibility criteria, inappropriate targeting of information about the programme by health practitioners and a general low level of awareness among families. Access to the programme was particularly challenging for women who did not speak English, had low literacy levels, were in low paid work or had fluctuating incomes. The potential impact was undermined by the rising price of food relative to voucher value. Access to registered retailers was problematic in rural areas, and there was low registration among smaller shops and market stalls, especially those serving culturally diverse communities.

**Conclusions:**

Our evaluation of the Healthy Start programme in England suggests that a food subsidy programme can provide an important nutritional safety net and potentially improve nutrition for pregnant women and young children living on low incomes. Factors that could compromise this impact include erosion of voucher value relative to the rising cost of food, lack of access to registered retailers and barriers to registering for the programme. Addressing these issues could inform the design and implementation of food subsidy programmes in high income countries.

## Background

Social and environmental barriers can inhibit a healthy diet and these barriers are significant for poor and marginalised women and children in high income countries [1-3]. Those who are poor are more likely to have diets that are energy dense and nutrient poor [4-6]. In particular, low income is associated with low intake of fruit and vegetables [7-11]. Good nutrition is particularly important during pregnancy, breastfeeding and early life to optimise the health of women and children [12,13]. Ensuring women have sufficient income during pregnancy to enable them to maintain a good level of health and nutrition has been suggested to be a key strategy for reducing health inequalities [14].

While there is some evidence that lack of nutritional knowledge and practical food preparation skills are contributory factors to poor diets in low-income families, structural barriers of affordability and access to fresh food are key [3,10,11,15]. It is difficult for low-income families to prioritise spending on food [16] in the context of a 25% increase in the price of fruit and vegetables and 12% rise in the cost of food generally [17] as well as falling living standards in recent years [18]. In 2012, 7% of households in the United Kingdom (UK) were reported to be unable to afford fresh fruit and vegetables and four million children and adults were estimated not to be eating a healthy diet [19]. The UK Family Food Survey 2011 [16] found that households in the lowest 20% of income were spending a higher proportion of their incomes on food than in 2007 but were buying less. The lowest income group purchased 15% less fruit and vegetables in 2011 compared to 2007, an average of 2.9 portions per person per day. This compares to an average of four portions of fruit and vegetables per person per day for all UK households [16].

As an example, a single pregnant woman under 25 years old expecting her first child and out of work receives £56.80 per week in income-based Jobseeker’s Allowance [20]. To eat a realistic, palatable diet that meets her nutritional requirements for pregnancy would cost her about £30.34 per week [21], up-rated by inflation [22]. This would mean spending 57% of her non-housing income on food. In reality, households in the lowest income decile in the UK spend on average 16.7% of their income on food, or £22.46 per person per week [23]. For the unemployed and those on low-incomes in the UK, household budgets are being further squeezed as a result of changes to the benefit and tax systems such as the introduction of a benefit cap [24]. It is currently estimated that benefit income for a couple with two children in the UK falls short of providing a minimum standard of living by £189 per week [25]. As low-income families spend a higher proportion of their budget on food, cuts to income and rising food prices are much harder to cope with, with large reductions in quality of food consumed as well disproportional impacts on spending on transport, health and recreation [17,26].

Food subsidy programmes are examples of policy approaches that aim to reduce financial barriers to healthy diets and tackle nutritional inequalities. Programmes such as the Special Supplemental Nutrition Programme for Women, Infants and Children (WIC), a federally funded programme in the United States (US), and Healthy Start in the UK, are directed towards women and young children. The WIC Programme in the US includes food supplementation, nutrition counselling and referral to health and social care services with the aims of safeguarding the health of disadvantaged women and children aged 1-4 years [27].

Healthy Start is a statutory means-tested food voucher programme that was introduced across the UK in 2006. Women who are at least ten weeks pregnant and families with children up to their fourth birthday can register for Healthy Start if they receive qualifying welfare benefits or qualifying tax credits with a household income of £16,190 or less (2013/14), or they are pregnant and aged under 18 irrespective of benefits or tax credits [28]. Those registered for Healthy Start receive vouchers which can be exchanged for fresh or frozen fruit and vegetables, plain cows’ milk or infant formula, and coupons for free vitamin supplements. The current voucher value is £3.10 per week for pregnant women and children between the ages of one and four, and £6.20 per week for children up to their first birthday. Application forms must be signed by a registered health professional. Healthy Start currently supports approximately 600,000 women and children in over 450,000 families in the UK [29]. The claim rate is around 80% of those eligible, in-line with the uptake of other means-tested benefits in the UK [30]. Of the 90% of vouchers that are redeemed, about 70% are spent in large supermarkets with the remainder spent in smaller retail outlets.

There is little evidence of the impact of food subsidy programmes on health outcomes for mothers and children. A recent systematic review [5] concluded that food subsidy programmes successfully increased the intake of targeted foods by 10-20% mainly for pregnant or postpartum women participating in WIC, and found a small but clinically relevant increase in mean birthweight (23–29 g) in two higher quality WIC studies. A small-scale, before and after study [31,32] found that women receiving Healthy Start food vouchers ate significantly more fruit and vegetables per day than those on the previous milk-based Welfare Food scheme. However, information on children’s intake is lacking in evaluations of food subsidy programmes [5,31]. The few economic studies that exist report findings from the US and have mixed results; while three studies reported that food vouchers were associated with increases in spending on fruit and/or vegetables [33-35], one showed a negative relationship [36] and two had mixed results or no association [37,38].

As well as impact, important dimensions of evaluating complex programmatic public health interventions such as food subsidy programmes include description of processes, contextual factors and qualitative data that might explain intervention effects [39]. Such evidence to inform the design and operation of food subsidy programmes is lacking, particularly from the perspectives of beneficiaries and health practitioners. Small-scale qualitative studies of WIC found that participants valued the inclusion of fresh fruit and vegetables and anticipated that this would increase intake [40]. For young single mothers, food vouchers were the only means by which they could afford to include fruit and vegetables in their diets [41]; and women highly valued the provision of free infant formula because it was an expensive product [42]. The only qualitative evidence of beneficiaries’ experiences of Healthy Start is from a rapid evaluation of a pilot in one UK region [43]. From interviews with beneficiaries, those authors concluded that there was a need for clearer information about the programme and about eligibility criteria; to reinforce healthy eating messages, health professionals should link with local services and encourage beneficiaries to take part in relevant practical, experiential activities locally; to ensure access and choice of retailers, additional information about registered retailers should be provided; and retail staff should be trained to minimise the potential embarrassment or stigma of using Healthy Start vouchers.

### Aims

We aimed to evaluate the Healthy Start programme in England from the perspectives of beneficiaries, potential beneficiaries and health practitioners and to focus on whether food vouchers can contribute to reducing nutritional inequalities for women and young children. We addressed two key questions:

Do Healthy Start food vouchers reach the families who need them?

Do Healthy Start food vouchers have the potential to improve nutrition for low-income women and young children?

## Methods

### Study design

This paper reports findings from a large multi-method evaluation that employed methods used previously by the research team [44,45] to elicit the views and experiences of a broad constituency of beneficiaries/potential beneficiaries, user representatives, practitioners, public health specialists, service managers and commissioners, and policy makers. The study comprised four key stages:

1. Focus group discussions with health practitioners involved in operationalising Healthy Start in their local areas

2. A national online consultation with health and social care practitioners, service managers, commissioners, and user and advocacy groups

3. Qualitative participatory workshops with low-income women from diverse backgrounds who were eligible or borderline eligible for Healthy Start, irrespective of whether they were registered for the programme. Additional focus group discussions and telephone interviews were conducted to include the views of women who did not speak English and those from Traveller communities

4. Cross-sectoral workshops with stakeholders including practitioners, service managers and commissioners, policy makers and advocacy groups.

Here we report findings from the first three stages relevant to the aims of this paper. The recommendations culminating from the cross-sectoral workshops, which were specific to England, will be reported elsewhere. User involvement, congruent with the principles of INVOLVE [46], was achieved through a key informant user panel comprising six women who were or had been registered for Healthy Start and who contributed their views on the design, conduct and interpretation of the findings throughout the study. The Yorkshire and the Humber – Humber Bridge NHS Ethics Committee approved the study [study reference number 11/YH/0272] and permission was granted by 12 NHS Trusts and one local authority to recruit participants in their localities. Informed written consent was obtained from all study participants taking part in focus group discussions and interviews.

### Setting

The qualitative work with practitioners and women was conducted in two regions of England; Yorkshire and the Humber, and London, selected because of their large and diverse populations. Localities within these regions were selected to provide opportunities to access different population groups and urban and rural contexts. The online consultation was circulated across stakeholder groups in England.

### Focus group discussions with health practitioners

Six focus groups discussions, lasting 40-60 minutes and attended by between six and eleven practitioners were held during March and April 2011; three in Yorkshire and the Humber (two in rural areas and one in a city) and three in London (all in inner city localities). Recruitment was facilitated by local Healthy Start leads. Each group was attended by two members of the research team, one to facilitate the discussion and the other to take detailed notes. The discussion guide covered all aspects of Healthy Start including perceived advantages and disadvantages of the programme, how eligible families are identified, experiences of the application process and awareness of how vouchers are used in the local area. Audio-recordings of the focus groups were used to add key information and illustrative quotes to the field notes.

### National online consultation

A web-based questionnaire was developed from the study aims and objectives, the findings of the practitioner focus groups, the views of collaborators and stakeholders and the key informant user panel. The questionnaire comprised nine sections and included closed and open questions and statements which respondents were asked to grade on a five-point Likert scale of ‘strongly agree’ to ‘strongly disagree’ with additional options of ‘don’t know’ and ‘not applicable’. The statements were drawn from those findings of the practitioner focus groups which had high consensus. Additional free text questions asked about barriers, strategies for improvements and examples of good practice. In July 2011, the web link to the questionnaire (using the platform Survey Monkey) was circulated by e-mail to extensive networks of practitioners, user representatives, strategic and operational managers, service commissioners and public health leads including all Regional Directors of Public Health, Regional Local Supervising Authority Midwifery Officers in England and professional associations (Royal College of Midwives, Community Practitioner and Health Visitor Association, Royal College of General Practitioners, Royal College of Paediatrics and Child Health, Royal Society for Public Health). The consultation was open for six weeks during July and August 2011.

### Evaluation of the views and experiences of women

#### *Recruitment*

An a priori sampling matrix guided purposive sampling to achieve maximum diversity of participants eligible for Healthy Start including women from specific groups such as teenagers, minority ethnic groups and those from urban and rural areas of high socio-economic disadvantage. Somali, Sylheti, Urdu and Polish speaking women were recruited to focus groups and women from Traveller communities participated in telephone interviews. We included women at all stages from pregnancy until their children were four years old, from any of the following categories:

● Women receiving vouchers

● Women who had received vouchers within the previous year

● Women who had recently applied for Healthy Start but were not yet receiving vouchers

● Women whose application for Healthy Start had been unsuccessful

● Women who thought they might be eligible for Healthy Start.

Although we expected that most participants would be women, men who were in any of the above categories or who wished to accompany their partners were welcomed. Recruitment was facilitated by health professionals, children’s centre staff and community workers. Venues were chosen that were familiar to women, easily accessible and in which women would feel comfortable. Wherever possible, women were drawn from pre-existing groups, as it was felt that knowing at least one other person would be less intimidating than being among strangers.

#### *Participatory workshops*

Participatory workshops use a combination of activities aimed at facilitating participation and the sharing of opinions and perspectives in an environment free from hierarchy and officialdom [47]. We chose this method to gain the trust of women from low-income and vulnerable groups, including those with little or no formal education. The workshops were facilitated by Food Matters, an NGO working on food policy issues with expertise in food access and participation, and were also attended by a researcher. Eleven workshops, six in Yorkshire and the Humber and five in London were held in children’s centres, community and housing association centres and a Young Person’s Education Centre between November 2011 and April 2012. Women were given an information sheet by recruitment facilitators a week before each workshop. The workshops, which lasted about two and a half hours, addressed a sequence of questions including; the purpose of Healthy Start and whether it achieved its aims, what recipients receive as part of Healthy Start and its impact on shopping, eating and health. Participants also completed a short demographic questionnaire and received a £20 honorarium. In two workshops, only one woman arrived on the scheduled day. In these workshops the facilitator used a modified version of the workshop activities to carry out an informal interview.

#### *Focus group discussions with women who did not speak English*

In March 2012, three focus group discussions were held to enable inclusion of women who did not speak English, as they would have found it difficult to participate fully in the English-language workshops. The focus groups, lasting 90 minutes, took place in a children’s centre in London and in a community centre and children’s centre in Yorkshire. A researcher facilitated the focus groups with the aid of interpreters. The interpreters were children’s centre staff or community health workers who knew the participants. The information sheet and consent form were translated by a professional translation service and checked for meaning by the interpreters. The topic guide was based on the same topics used for the participatory workshops. Each focus group was audio-recorded and key points extracted.

#### *Telephone interviews with women from Traveller communities*

Specialist health visitors working with Traveller communities in a city in Yorkshire found it difficult to recruit women from those communities to a participatory workshop. However three women were willing to share their experiences of the Healthy Start programme in one-to-one telephone interviews. The telephone interviews, lasting 30 minutes and addressing the same key topics as the workshops and focus groups took place in April 2012. The researcher took notes of key points from the interviews.

### Analysis

The framework method [48] was used to analyse the research material. A framework of nine themes, as listed below, was derived from the different aspects of the programme and the aims of the study and agreed by the research team.

1. Importance of Healthy Start

2. Awareness of the programme

3. Opportunities for providing health-related and lifestyle information

4. Eligibility

5. Application process

6. Using Healthy Start vouchers

7. Healthy Start vitamin supplements

8. Health Start and infant feeding

9. Education and training for healthcare practitioners

All qualitative research material was coded deductively according to the nine themes. Within each theme, inductive/open coding was used to identify subthemes. The lead researcher (AM) was involved in coding data from all stages of the study with co-analysis and discussion of interpretation with different members of the research team for the different stages of the study. The data from the different methods and groups of participants were triangulated to enhance rigour and to identify commonalities and differences. All the research team discussed the interpretation of differences between the multiple sources of data. Analysis of quantitative data from the online consultation comprised descriptive statistics.

## Results

### Participants

#### *Focus group discussions with health practitioners*

As shown in Table 1, 49 practitioners, representing a range of disciplines and roles in respect of Healthy Start and who worked with vulnerable groups, participated in focus groups.

**Table 1:** Practitioner focus group participant roles

	**Yorkshire and the Humber**			**London**			**Totals**
**Characteristics of study site**	**Rural**	**Rural**	**Urban**	**Urban**	**Urban**	**Urban**	
**Health Visitor**	2		1	2	2	2	9
**Public Health Specialist**	1	1	1	1	1	2	7
**Midwife**	1	1	2			2	6
**Administrator**	1		2	1	1	1	6
**Infant Feeding Specialist**	1	1				1	3
**Support Worker**	1		2		1		4
**Service Manager**	2	1	1	1			5
**Nursery Nurse**	1			1	1		3
**Children’s Centre Manager**	1	1					2
**Other**		1	1	2			4
**Total**	11	6	10	8	6	8	49

#### *National online consultation*

The questionnaire was completed by 620 respondents representing a wide range of roles and from all English regions (Table 2).

**Table 2:** Summary of national online consultation participant roles

	**N**	**%**
**Health Visitor**	217	35.1
**Midwife**	134	21.6
**Public Health Specialist**	53	8.6
**Dietician**	26	4.2
**Infant Feeding Specialist**	22	3.6
**Support Worker**	20	3.2
**Early Years’ Practitioner**	11	1.8
**Nutritionist**	9	1.5
**Nurse**	8	1.3
**Paediatrician**	8	1.3
**Voluntary Sector Supporter/User Representative**	8	1.3
**Administrator**	7	1.1
**General Practitioner**	7	1.1
**Service Commissioner**	7	1.1
**Other**	82	13.2
**Total answered question**	619	

#### *Evaluation of the views and experiences of women*

Altogether, 109 women and four men took part in this phase of the study. The number of participants attending the three elements was: participatory workshops (n = 81), focus group discussions for women who did not speak English (n = 25) and telephone interviews with women from Traveller communities (n = 3). Table 3 shows the characteristics of the 109 participants who completed the demographic questionnaire. Only 12 women were aged 20 years or under. Over half of the participants were from minority ethnic backgrounds and about 40% reported that English was not their first language. Most participants were unemployed (67%) and a third had no educational qualifications. Overall, 58% of participants were in receipt of Healthy Start vouchers, a further 12% had received them but were no longer eligible (mostly because their child had reached his/her fourth birthday), 18% were unsure of their eligibility and 5% reported they were not eligible.

**Table 3:** Evaluation of the views and experiences of women: participant characteristics

**Participant characteristic**	**All N (%) N=109**	**Participatory workshops N=81**	**Focus groups (non-English speaking women) N=25**	**Telephone interviews (Travellers) N=3**
**Gender**				
Female	105 (96.3)	77 (95.1)	25 (100)	3 (100)
Male	4 (3.7)	4 (4.9)		
**Age**				
≤20	12 (11.1)	12 (14.8)		
21-30	56 (51.3)	47 (57.9)	9 (36)	
31-40	34 (31.2)	19 (23.3)	12 (48)	3 (100)
>40	4 (3.7)	1 (1.2)	3 (12)	
missing	3 (2.8)	2 (2.5)	1 (4)	
**Ethnic background**				
**White British**	43 (39.4)	43 (53.1)		
**White other**	8 (7.3)	3 (3.7)	2 (8)	3 (100)
**Asian**	30 (27.5)	15 (18.5)	15 (60)	
**Black**	20 (18.3)	13(16)	7 (28)	
**Arab**	1 (0.9)	1 (1.2)		
**Mixed**	2 (1.8)	2 (2.5)		
**Other**	5 (4.6)	4 (4.9)	1 (4)	
**No. of children**				
**0**	4 (3.7)	4 (4.9)		
**1**	30 (27.5)	27 (33.3)	3 (12)	
**2**	24 (22)	22 (27.2)	1 (4)	1 (33.3)
**3**	27 (24.8)	21(25.9)	6 (24)	
**4**	9 (8.3)	2 (2.5)	6 (24)	1 (33.3)
**≥5**	15 (13.8)	5 (6.1)	9 (36)	1 (33.3)
**Age of youngest child in months**				
**0-11**	39 (35.9)	33(40.7)	6 (24)	
**12-23**	17(15.5)	13 (15.9)	4 (16)	
**24-35**	28 (25.7)	20 (24.7)	6 (24)	2 (66.7)
**36-47**	12 (11)	8 (9.8)	3 (12)	1 (33.3)
**≥48**	9 (8.2)	4 (4.9)	5 (20)	
**Missing**	4 (3.7)	3 (3.7)	1 (4)	
**Highest educational qualification**				
**None**	36 (33)	24 (29.6)	9 (36)	3 (100)
**GCSE D-G**	27 (24.8)	20 (24.7)	7 (28)	
**GCSE A-C**	19 (17.4)	17 (21)	2 (8)	
**A level**	12 (11)	11 (13.6)	1 (4)	
**Degree**	6 (5.5)	6 (7.4)	0	
**Missing**	9 (8.3)	3 (3.7)	6 (24)	
**Employment status**				
**Maternity leave**	6 (5.5)	6 (7.4)	0	
**Student**	12 (11)	10 (12.3)	2 (8)	
**Employed full-time**	2 (1.8)	2 (2.5)	0	
**Employed part-time**	6 (5.5)	3 (3.7)	3 (12)	
**None**	73 (67)	53 (65.4)	17 (68)	3 (100)
**Missing**	10 (9.2)	7 (8.6)	3 (12)	
**Partner’s employment**				
**Student**	2 (3.8)	2 (2.5)	0	
**Employed full-time**	11 (1.8)	8 (9.9)	3 (12)	
**Employed part-time**	12 (11)	4 (4.9)	8 (32)	
**None**	28 (25.7)	18 (22.2)	8 (32)	2 (66.7)
**Missing**	56 (51.4)	49 (60.5)	6 (24)	1 (33.3)
**First language English**				
**Yes**	62 (56.9)	57 (70.4)	2 (8)	3 (100)
**No**	45 (41.3)	23 (28.4)	22(88)	
**Missing**	2(1.8)	1 (1.2)	1 (4)	
**Pregnant**				
**Yes**	14 (13.3)	11 (13.6)	2 (8)	1(33.3)
**No**	91 (83.5)	67 (82.7)	22 (88)	2 (66.7)
**Missing**	4 (3.7)	3 (3.7)	1 (4)	

### Overview of themes

To answer our research question concerning the potential contribution of food vouchers to reducing nutritional inequalities for women and young children, we present relevant findings under two major themes. The first is *accessibility of Healthy Start* which subsumes the framework -themes of eligibility, awareness of the programme, and the application process. The second is the framework theme *using food vouchers,* which includes the sub-themes of *influence of Healthy Start vouchers on food choices* and *accessing retail outlets.* Direct quotes from participants are shown in italics.

### Accessibility of healthy start

#### *Eligibility*

There was consensus across participants that the eligibility criteria were clear for families who were in receipt of qualifying welfare benefits. Half of respondents to the online consultation thought that the criteria were about right while a third thought more women should be eligible. However, both women and practitioners said that the eligibility criteria relating to qualifying tax credits were confusing and that the household income threshold for families receiving tax credits was too low and discriminated against those in low paid work. Comments included:

When I was working I was worse off. Now I am on benefits I’m better off. I get vouchers and other support. (London workshop participant)

It is no good having a threshold of £16,000 because everything has gone up – VAT, petrol, but the threshold hasn’t gone up has it? So people on a low income have to cut back everything (Sylheti-speaking focus group participant)

Women also reported that the ‘annual income’ test created problems for families moving in and out of low paid work or with variable earnings from self-employment.

The system (Healthy Start) is not successful because I have five kids. My husband is self-employed-sometimes he has loads of work and sometimes we have to scrimp and sometimes he has no work. I want to be able to access the vouchers when my husband has no work (Yorkshire and Humber workshop participant, rural).

Some women reported that understanding eligibility could be complex if their eligibility for welfare benefits or tax credits changed following the baby’s birth. This was especially confusing for women under 18 years old because Healthy Start is a universal benefit for this group during pregnancy but is means-tested after birth and following their 18^th^ birthday.

I get working and child tax credits. I did get the vouchers when I was pregnant but after the baby was born they said the scheme was not available anymore. I don’t know why (Urdu-speaking focus group participant).

Many women thought that eligibility should extend to the child’s fifth birthday and among non-English speaking participants, there was consensus that the programme should extend beyond five years to establish good eating habits in children. Many practitioners were concerned that those with uncertain immigration status (e.g. seeking asylum), some of the most vulnerable groups of women and children in the UK, were not eligible for Healthy Start food vouchers.

#### *Awareness of healthy start*

A key factor in whether eligible women register for Healthy Start and receive food vouchers is their awareness of the programme. Only a quarter of respondents to the online consultation thought that the women they saw were already aware of their eligibility for Healthy Start, highlighting the importance of practitioners giving women Healthy Start information. However, not all women were told about Healthy Start by their midwife or health visitor and a few women had not found out about Healthy Start until their child was over two years old. Women did not appear to be aware of the scheme from other sources such as leaflets on benefits and tax credits or through government helplines when applying for benefits and tax credits. Several women had never heard of Healthy Start or knew very little about it, even if they had been in contact with health professionals during pregnancy and their child’s first four years. This was particularly evident among women who did not speak English. Practitioners corroborated the difficulty of publicising Health Start to women who do not speak English and those with poor literacy, because of the lack of information in languages other than English or in non-written formats.

I’m six month pregnant and until today I didn’t know I was able to get Healthy Start (London workshop participant)

I see different health visitors and sometimes it’s a language barrier and they are coming for a home visit and the most things they are asking is what are the children eating and what kind of food can I afford. They don’t give information about what benefits we are entitled to (Polish-speaking focus group participant).

Practitioner focus group participants and online consultation respondents identified that another key barrier to providing effective information about Healthy Start was the amount of pregnancy information that is given to women at the first antenatal contact resulting in ‘information overload’. Consequently some busy health practitioners, usually community midwives and health visitors (public health nurses), targeted information to those they judged to be eligible. However, many participants were concerned that eligible families were missed because incorrect assumptions were made about their economic circumstances or because their circumstances changed - just over half of consultation respondents agreed that they could easily identify women who were eligible, and some were reticent about asking women about their financial circumstances. In addition, while most practitioners suggested their responsibility was to provide information about the programme, a minority viewed their role as gatekeepers of eligibility, expressing concern that some women may abuse the system. Many practitioners recommended that all women should be informed about Healthy Start and that awareness among the general population should be raised.

Biggest issue we are having is to differentiate between those not working [from those who are working] – all health professionals feel the same, nurses, doctors etc. – having to have the conversation (London practitioner focus group participant).

#### *Application process*

According to the reports of women and practitioners, the barriers to registering for Healthy Start described in the previous sections were exacerbated by a cumbersome application process. Women who did not speak English or with poor literacy described problems with completing the application forms. They sought help from friends and family, bilingual health and social care practitioners, children’s centre staff or other community services. While 50% of online consultation respondents agreed there was support available to help women complete application forms, only 20% thought there was such help for women who were not fluent in English. One Urdu-speaking participant brought a letter from the Healthy Start issuing department to the focus group because she could not understand it. Several women described applying once and being refused and applying a second time and being accepted and they did not understand the reasons for this. Some women assumed that if they did not hear from the issuing department it meant that they were ineligible whereas others had followed up their claims successfully.

I got the information and filled in the forms but I never got a reply back. There was no point applying again and again and I couldn’t ring to ask because of the language barrier. (Urdu-speaking focus group participant)

### Using healthy start vouchers

#### Influence of healthy start vouchers on food choices

Women taking part in this study suggested that food vouchers made a difference to their families’ shopping and eating habits.

In the week the vouchers come we can eat vegetables (telephone interviewee from Traveller community)

The majority of women reported that the vouchers enabled them to buy better quality and a greater variety of fruit and vegetables. One woman from a Traveller community described how, when she no longer received vouchers, she could not experiment with different types of fruit because she could not afford waste. Many women said they would buy similar amounts of milk, fruit and vegetables even if they did not get the vouchers; however the vouchers helped them to manage better financially. Others reported that they bought less fruit and vegetables once the vouchers ceased. The vouchers were also said to provide a reminder of the need to eat a healthy diet, and to help establish good habits for the future.

I am in the habit of shopping for fruits and vegetable so I think I’ll carry on. Get your kids used to it and demand it of you (London workshop participant)

Several young mothers said that Healthy Start provided them with resources for food to which they would not otherwise have access. One young mother who lived with her parents said the food vouchers were the only income she had. A pregnant teenager described the impact of the vouchers on her diet and health.

I used to live on junk food - now I’m eating healthy. Without vouchers I wouldn’t buy fruit and veg. (Yorkshire and Humber teenage workshop participant, urban)

While many women and practitioners felt that the vouchers made a difference to family budgets, there was consensus that the voucher value should keep pace with the rising cost of food, so that the potential to improve family diets was not undermined.

A few women suggested that the money would be better added to welfare benefits instead of provided as food vouchers. However, others thought this risked the money not being spent on children. One participant from a Traveller community asked if she could be paid the £20 honorarium in Healthy Start vouchers. This suggested the vouchers had more value to her than cash.

Healthy Start appeared to have greater influence on diet and nutrition among mothers who breastfed exclusively, because they could spend the vouchers on fruit, vegetables or plain cows’ milk. Women who were formula feeding their babies spent all their vouchers on formula and commented that the vouchers did not cover the whole cost.

Having vouchers for formula doesn’t influence the decision to not breastfeed but if it’s not going well it means that having a way to help with the cost of formula takes away the worry about how to feed your baby (Yorkshire and the Humber workshop participant, rural)

There were different opinions among practitioners; some felt that allowing vouchers to be used for infant formula incentivised women to formula feed whereas others thought that women should have access to resources to feed their infants regardless of their infant feeding decisions. Some of the latter group felt the value of the vouchers should be increased to cover the entire cost of infant formula.

We are not health police and if mothers decide to formula feed that is their choice and they should have access to resources to feed their babies (London practitioner focus group participant).

#### *Accessing retail outlets*

Most women spent their vouchers at major supermarkets; for many this was the most convenient and cost-effective option. However, many women and practitioners highlighted problems with the range and location of retail outlets that accepted Healthy Start vouchers. Location was a particular problem for families living in rural areas where visiting a supermarket could involve a lengthy and costly journey. Some women and practitioners suggested the vouchers should be valid for online shopping as an alternative.

Rural area, some women have to travel up to 11 miles, often by bus, to spend vouchers - not cost effective. No small ‘corner shops’ will exchange locally (online consultation respondent).

Many women would have preferred to spend their vouchers in small shops or market stalls but found most were not registered to redeem the vouchers. Women from minority ethnic backgrounds suggested they could not find culturally acceptable fruit and vegetables in supermarkets and that local shops and market stalls were not registered.

Shops [small independent retailers] would not want to get involved in the form-filling or probably they are not aware of the scheme. If we don’t know about it how would the shopkeepers know? (Sylheti-speaking focus group participant)

A related problem for many women was not knowing which retail outlets in their area were registered. Women were reluctant to ask shopkeepers because asking the question identified them as being poor and receiving benefits. This stigma could also be experienced in supermarkets as some women described feeling judged by retail staff or other customers.

Practitioners were aware of these problems and while some were wary of the workload involved, others had successfully worked with local retailers to encourage them to register for Healthy Start.

It would be good if cheaper market stalls could take vouchers but would it be a lot of administration? It would make such a difference (London practitioner focus group participant).

Where there has been community buy-in for food outlets there have been real positives e.g. work with local market fruit and veg. stallholders are now able to take vouchers – active promotion and marketing enables the scheme to work better (Yorkshire and the Humber practitioner focus group participant, rural).

## Discussion

Our results show that both beneficiaries and practitioners valued the contribution of Healthy Start food vouchers to improve the diets of pregnant women, mothers and young children under four years-old in low-income families. Women reported that food vouchers increased the quantity and range of fruit and vegetables eaten by them and their children. This was said to improve the quality of family diets while receiving food vouchers and to establish good habits for the future. The influence of food vouchers appeared to have particular salience for teenage pregnant women who may not otherwise have had autonomy or access to resources to buy nutritious food. Thus our study supports findings from WIC that food vouchers increase intake of fruit and vegetables [5,41].

However participants highlighted concerns that could compromise the potential for Healthy Start vouchers to reduce nutritional inequalities. For those eligible for the programme, barriers to registration meant that not all those who could benefit from food vouchers received them. These barriers included complex eligibility criteria that are difficult for women and health practitioners to understand, inappropriate targeting of information about the programme by health practitioners to those they judge to be eligible and low level of awareness of the programme among the general population. Additionally a complex application process led to some potential beneficiaries losing out and caused delays in receiving time-limited vouchers for others. Complex bureaucracy is a feature of means-tested benefits and often leads to low uptake and frequent administration errors as experienced by many of the participants in our study [49].

From our study, access to the programme was particularly challenging for several groups of eligible women. Women in low paid work with a household income of £16,190 or less per annum (2013/14 figures) receiving tax credits may be eligible for food vouchers but may not be identified as such by health practitioners and consequently not signposted to apply. Relying on busy health professionals to signpost women to a welfare entitlement means they unwittingly become gatekeepers. Women who do not speak English and those with low literacy levels have particular challenges in finding out about the programme and negotiating the application process. Administration processes for Healthy Start are not sufficiently responsive to accommodate the needs of families whose household income fluctuates. Those who may be most financially and nutritionally vulnerable, such as women with uncertain immigration status, are not eligible for Healthy Start although they may be entitled to a small amount of financial support administered by the National Asylum Seeker Support Service. The income threshold of £16,190 excludes many families who are struggling financially to maintain a healthy diet [23]. It is estimated that two parents with two children need to earn £18,400 each to maintain a minimum standard of living [25]. In comparison the threshold for WIC in the US is more generous and increases according to family size [50].

A key issue for those in receipt of food vouchers was that the potential impact was undermined by the rising price of food relative to voucher value. Food vouchers are likely to become even more important to low-income families in the context of changes to welfare benefits in the UK. Welfare benefits have historically risen in line with inflation; however, current government plans are to limit future rises in welfare benefits at 1% per year until 2016 [51]. Unless inflation is below 1%, families receiving benefits will see the cost of food rising faster than their incomes. This will increase the importance for pregnant and breastfeeding women and young children of having extra support to buy fruit, vegetables and milk. If the value of the vouchers does not keep pace with the rising cost of food, the nutritional safety net will be eroded. Family budgets will be further squeezed by the benefit cap introduced in 2013 and set at £500 per week for a couple or lone parent regardless of family size, thereby disproportionately affecting children in larger families.

The effectiveness of Healthy Start in addressing nutritional inequalities was also affected in some areas by the limited range and location of retail outlets registered for the programme. Practical challenges were identified such as difficulties or expenses of transport especially in rural areas, and the low registration among smaller shops and market stalls, particularly those serving culturally diverse communities. Understandably, health practitioners were wary about the workload involved if they took responsibility for addressing these issues. This suggests a need for a broader policy approach involving the retail sector [52]. In addition, some women felt stigmatised because the vouchers identified them as being poor.

The inclusion of infant formula as an allowable product, while providing a nutritional safety net for low-income families, may contribute to nutritional inequalities by reducing the proportion of voucher value that is available to spend on fruit and vegetables. Similarly, Holmes et al [42] suggested that the provision of free infant formula as part of WIC contributes to low breastfeeding rates in the US. Two large-scale UK surveys reported lower rates of breastfeeding in in the Healthy Start sub-sample compared to the UK sample [53,54]. In the Infant Feeding Survey 2010 [54], breastfeeding rates for women registered for Healthy Start were lower than both those who thought they were eligible but were not registered and mothers who had never worked. While confounding variables such as age, socio-economic status and ethnicity may partially explain this finding, the relationship between receiving Healthy Start food vouchers and breastfeeding is worthy of further investigation.

There are challenges to comparing our findings to evaluations of WIC in the US. In terms of its impact, WIC has been shown to increase mean birthweight by a small but clinically significant amount [5]. However, among key differences compared to Healthy Start, WIC includes a wider range of food items, has more generous income-related eligibility criteria, continues until the child’s fifth birthday and includes nutrition counselling. In spite of this, there are reports that, during the current economic crisis in the US, enrolment in WIC is falling. Conversely, uptake of the Supplemental Nutritional Assistance Programme (SNAP) is increasing. Among the complex reasons for this it has been suggested that families with young children are turning to SNAP because enrolment is less bureaucratic and SNAP is more generous than WIC [55]. This supports our findings that barriers to registration and voucher value could undermine the impact of food voucher programmes.

### Methodological considerations

We believe this is the most comprehensive evaluation of a food subsidy programme from the experiences of those both sides of the frontline i.e. beneficiaries and health practitioners. Strengths of this research include the range of methods used and the wide range of participants comprising 109 women, four men and 725 practitioners, service managers, commissioners, policy makers and members of advocacy groups. The high degree of consistency of views from these diverse methods and participants provides some confidence in the validity of the findings. Our flexible, purposive approach to sampling guided by an a priori sampling framework resulted in recruitment of diverse participants. Consequently we were able to explore the experiences and barriers for those registered for the programme as well as those who were eligible but not registered and those who were borderline eligible. We were particularly successful in recruiting participants from minority ethnic backgrounds and those who did not speak English, groups who are frequently under-represented or excluded from studies. The group that proved most challenging to recruit were under-18 year olds and although 12 women (11% of our sample of women) were aged 20 or under we had aimed to conduct three participatory workshops with up to 30 women from this age group. Collaborating with a NGO that had experience in working on food policy issues and with expertise in food access and participation was a successful strategy for gaining the trust of participants and fostering an informal atmosphere in which participants felt able to contribute their views.

The findings are limited in that they reflect self-report of experiences and behaviours. The study did not aim to assess dietary intake to confirm reported increased intakes of fruit and vegetables. Further research is needed to measure the impact of food vouchers on nutritional intakes and health outcomes and further comparative research is needed to assess the effectiveness and cost-effectiveness of Healthy Start food vouchers. Although the findings of this study are applicable to the Healthy Start programme in England, insights from the experiences of the participants may be relevant to other food subsidy programmes in high income countries.

Forthcoming papers will report on the evaluation of the provision of vitamin supplements as part of the Healthy Start programme, and the recommendations for the operation of the Healthy Start programme in England culminating from the cross-sectoral workshops.

## Conclusion

Our evaluation of the Healthy Start programme in England suggests that a food subsidy programme can provide an important nutritional safety net and potentially improve nutrition for pregnant women and young children living on low incomes. The Healthy Start vouchers guaranteed access to at least some vegetables, fruit and/or milk in the context of a weekly food budget under severe pressure. However, insight from the experiences of beneficiaries, potential beneficiaries and practitioners highlighted several factors that could compromise the impact of food vouchers. A key problem was the erosion of voucher value relative to the rising cost of healthy food. Two issues related to access to retailers; barriers included distance to appropriate retail outlets for families living in rural areas and low registration among smaller shops and market stalls serving culturally diverse communities. There were barriers to accessing the programme for those least likely to be registered; mainly those who do not speak English, those with literacy problems, those in low paid work and those whose income fluctuates. Our participants felt that the eligibility criteria excluded many who could benefit including those with uncertain immigration status and those in work but who were just above the income threshold. Consideration of these issues could inform the design and implementation of food subsidy programmes in high income countries, and help to address nutritional inequalities.

## Competing interests

The authors declare that they have no competing interests.

This is an independent report commissioned and funded by the Policy Research Programme in the Department of Health, UK. The views expressed in the publication are those of the authors and not necessarily those of the Department of Health.

## Authors’ contributions

AM conceived and designed the study, collected, analysed and interpreted the data, and drafted the manuscript, JMG conceived an designed the study, collected, analysed and interpreted the data, VW designed the study and collected, analysed and interpreted the data, JM collected, analysed and interpreted the data, FM collected and analysed the data, JFR conceived and designed the study, MJR conceived and designed the study and interpreted the data. All authors revised the manuscript for intellectual content and approved the final version.

## Pre-publication history

The pre-publication history for this paper can be accessed here:

http://www.biomedcentral.com/1471-2458/14/148/prepub
